# Effectiveness of Shock Wave Therapy as a Treatment for Spasticity: A Systematic Review

**DOI:** 10.3390/brainsci11010015

**Published:** 2020-12-24

**Authors:** Isabel María Martínez, Nuria Sempere-Rubio, Olga Navarro, Raquel Faubel

**Affiliations:** 1 Faculty of Physiotherapy, University of Valencia, 46010 Valencia, Spain; isabelmariamarttinez@gmail.com; 2 Asociación de Familiares y Enfermos de Parkinson de Villarrobledo, Centro de Rehabilitación Neurológica (Neurovilla), 02600 Albacete, Spain; 3 Department of Physiotherapy, University of Valencia, 46010 Valencia, Spain; raquel.faubel@uv.es; 4 Research Unit in Clinical Biomechanics (UBIC), Department of Physiotherapy, University of Valencia, 46010 Valencia, Spain; 5 Department of Nursery, Catholic University of Valencia, 46001 Valencia, Spain; olga.navarro@ucv.es; 6 Isntituto ITACA, Universitat Politècnica de València, 46022 València, Spain; 7 Joint Research Unit in Biomedical Engineering, IIS La Fe-Universitat Politècnica de València, 46026 Valencia, Spain; 8 PTinMOTION, Physiotherapy in Motion, Multispeciality Research Group, Department of Physiotherapy, Universitat de València, 46010 València, Spain

**Keywords:** muscle spasticity, spasticity, extracorporeal shockwave therapy, shock wave

## Abstract

Background: The purpose of this study was to collect and analyse the available scientific evidence on the effectiveness of shock wave therapy as a treatment for spasticity. Methods: the search was performed in the following databases: PubMed, PEDro, Cochrane, Embase, and the Virtual Health Library. All publications from November 2009 to November 2019 were selected that included a sample of patients with spasticity and prior suspension of botulinum toxin, to whom shock wave therapy was applied. The methodological quality of the articles was evaluated using the Jadad scale and the pyramid of quality of scientific evidence. Results: 25 studies involving 866 participants with spasticity were selected. The results obtained suggest that shock wave therapy appears to be effective in reducing spasticity levels irrespective of the age of the participants, the type of injury, and the tool used to measure the effect. Conclusions: shock wave therapy reports evidence of improvement in motor function, motor impairment, pain, and functional independence, applied independently of botulinum toxin. However, due to the heterogeneity of the protocols, there is no optimum protocol for its application, and it would be appropriate to gain more high-quality scientific evidence through primary studies.

## 1. Introduction

Spasticity is a frequent complication in neurological diseases and a great clinical challenge [1], which causes a high burden of care and economic implications [2]. The concept has been known, since the 19th century, as a resistance to passive movement [3], and it has been defined as a “a motor disorder characterized by a velocity-dependent increase in tonic stretch reflexes with exaggerated tendon jerks, resulting from hyperexcitability of the stretch reflex, being one of the signs of upper motor neurone syndrome (MNS)” [4]. The most common complications related to spasticity include: chronic neuropathic pain, sensory disorders, bone deformities with demineralisation, severe muscle spasms, fibrosis of muscle fibres, and muscular atrophy with rheological changes [1,5,6,7]. Furthermore, it interferes with daily life by impairing physical capabilities (restricted range of joint movement, loss of dexterity, impaired balance and walking) [8,9], which, together with the emotional impact (on character, mood, and self-esteem), can lead to social isolation [10]. In Spain, approximately 10 in every 1000 inhabitants live with this multi-factor clinical condition [2].

Its prevalence is linked to associated pathology; it is estimated that it affects around 20–40% of survivors of stroke after 12 months [1,6,9,11,12,13,14,15], 60–90% of people with multiple sclerosis [9], and 80% of patients with cerebral palsy (CP) [5,16]. Between 60 and 78% [2] of people with spinal cord injuries and around 13–20% of people who suffer head trauma present spasticity [2]. Selection of a treatment plan is complicated, and different therapeutic strategies are used to modulate muscle tone [8,17]. Some of the therapeutic approaches include physiotherapy [6,12,14,18,19], antispasmodic medications [5,6,8,19,20], and orthopaedic surgery [10,13,14]. Both pharmacology (dantrolene, benzodiazepine, gabapentin, nabiximols, intrathecal baclofen, tizanidine, clonidine, phenol injections, ethanol, and botulinum toxin (BTA)) and surgery (tenotomies, tendon transfers, neurectomies, and rhizotomies) are used in combination with other forms of treatment for inter-disciplinary rehabilitation [9]. Physiotherapy plays a vital role in clinical management of spasticity, using physical agents and different types of methods and techniques [12,19,21], such as muscular stretching, cryotherapy, taping, splints and orthoses, ultrasounds, vibration therapy, electrical stimulation, transcutaneous electrical nerve stimulation, and dry needling for hypertonia and spasticity (DNHS) [21]. However, there is a need for new methods of non-invasive treatment for spasticity [5,13], such as shock wave therapy, a recent method that is reversible and non-invasive [1,6].

Shock wave therapy is defined as a sequence of single sonic pulses, characterized by high peaks and a rapid increase in pressure and short duration [7,11,14,19,20,22], which act through direct modulation of the rheological properties of the muscle tissue; it seems that the vibration breaks the functional link between the actin and the myosin, reducing the rigidity of the connective tissue [16,19]. The types of treatments can be divided into focused or radial shock waves. The total number of sessions vary, from a minimum of 1 session up to more than 20 sessions. The injections administered can be between 500 and 4000; the energy can range between 0.03 mJ/mm^2^ -1.5 bar and 3.5 bar, and the frequency varies between 4 Hz and 10 Hz [1,11,14,16]. In recent years, shock waves have been tested on spasticity, and were shown to be safe and effective in reduction [13,18,23], with few observed side effects, these being transitory [14,24].

Previous studies have determined its impact on patients with various neurological pathologies, such as stroke [1,6,12,13,14,15,18,19,23,25,26,27,28,29,30], CP [5,8,16,17,20,22,24,26,31], and spinal cord injuries [26]. The bibliography features recent reviews [11,32,33,34,35], which analysed its effectiveness, although they all include patients who have received BTA prior to the study. As use of BTA has damaging long-term effects [14], the objective of this review is to collect and analyse the available scientific evidence on the effectiveness of shock wave therapy as a treatment for spasticity, applied independently of other treatments, such as BTA.

## 2. Materials and Methods

The study was carried out according to the PRISMA recommendations for systematic reviews and meta-analysis [36].

### 2.1. Literature Search Strategy

The systematic search was carried out in PubMed, PEDro, Cochrane, Embase, and the Virtual Health Library. The following search equation was used: (espasticidad OR “muscle spasticity” OR spasticity) AND (“ondas de choque” OR “extracorporeal shock wave therapy” OR shockwave OR “shock wave”) for PubMed and the Virtual Health Library. The following keywords were used in the other databases: muscle spasticity, spasticity, extracorporeal shockwave therapy, and shock wave. A manual search was also carried out, which included the references of the articles found and related articles.

### 2.2. Selection of Studies

Studies published in any language over the last 10 years, which carried out intervention on spasticity using shock waves, and showed results for the effect of the shock waves on the (physical or psychological) symptoms or functionality, were included. Studies were excluded if interventions with shock waves were applied in combination with interventions from other disciplines (occupational therapy or speech therapy), as were studies that included participants who had received BTA.

### 2.3. Data Extraction

Data were extracted using a standardised data collection sheet from Microsoft Excel. Data regarding study design, sociodemographic characteristics of participants, protocol used, intervention carried out on the control group, and the variables of the results, were extracted (Table 1).

### 2.4. Analysis of Methodological Quality

Methodological quality was assessed using the Jadad scale for the randomised control trials (RCTs) [37]. This scale is made up of a total of 5 items, giving a score between 0 and 5 to show low quality (0–2), acceptable quality (3), and high quality (4–5). Studies with other designs were evaluated through critical evaluation using the pyramid of evidence [38]. The Before and After Quality Assessment (BAQA) tool was used for the quality assessment of pre–post quasi-experimental studies [39].

## 3. Results

### 3.1. Results of the Literature Searches and Selection of Studies

**Figure 1:**
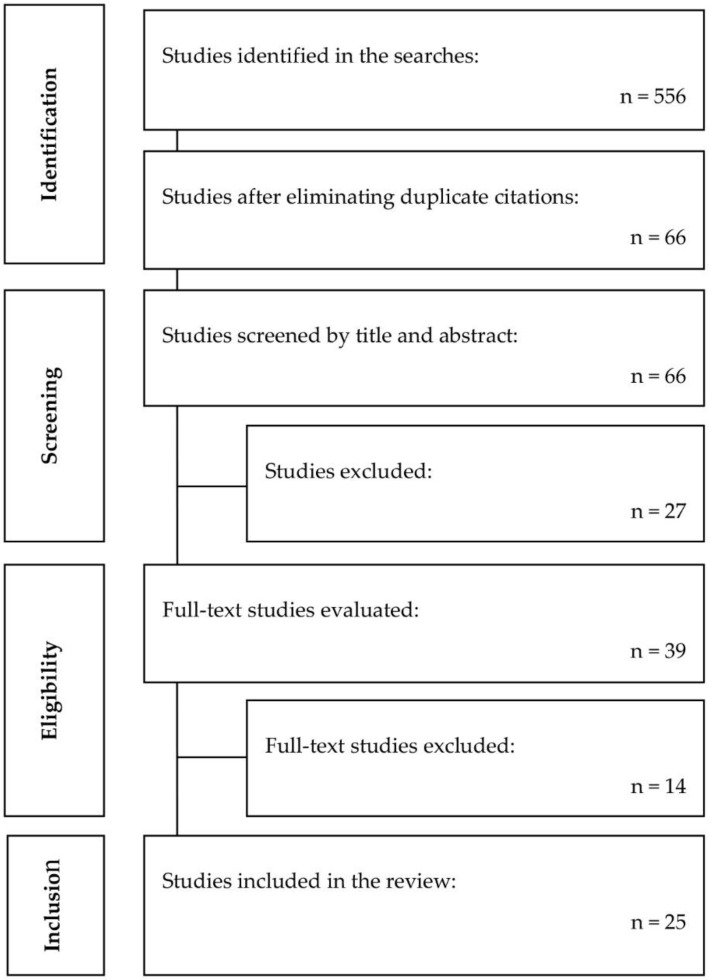
Flow diagram of the process of literature search and selection of studies included in the review.

As shown in the article selection flow diagram (Figure 1), the total number of articles identified was 556: 168 from PubMed, 34 from PEDro, 107 from Cochrane, 173 from Embase, 226 from the Virtual Health Library. After duplicates were eliminated, 66 references were analysed by reading the title and abstract, and 41 of these were eliminated as they did not meet the inclusion criteria. After critical reading of the entire text, a total of 25 studies were selected for inclusion in this review, 14 were randomised controlled trials (RCTs).

### 3.2. Characteristics of the Patients in the Studies Included in the Review

**Figure 2:**
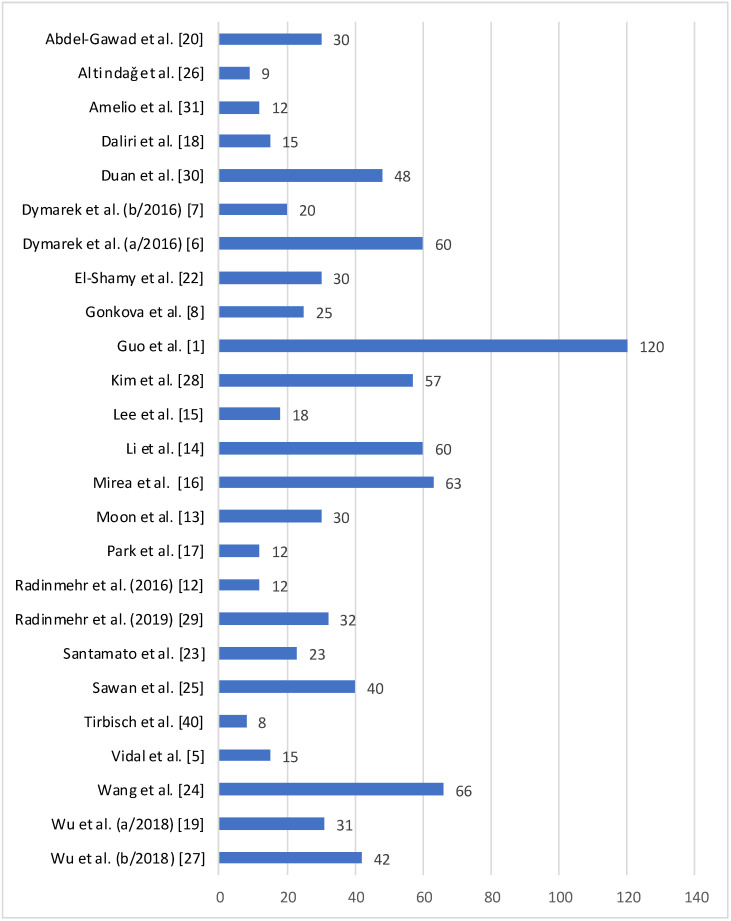
Sample size of studies included in the review.

From all of the studies reviewed, the sample consisted of 866 patients and the sample size of the different studies ranged from 8 [40] to 120 [1] participants, with an average size of 35.12 participants (Figure 2. Regarding the age of the people included in the study, the average age was 40.02 years, and ages ranged from 26.9 months [24] to 84.8 years [25]. The study patients presented different pathologies: 619 had at least one episode of stroke, 243 were diagnosed with CP, and the other 4 suffered from spinal cord injuries.

### 3.3. Characteristics of the Studies Included in the Review

Of the 25 articles included in this review, six used shock wave therapy in comparison with a programme of conventional rehabilitation [20,22,24,26,30,40], another ten applied shock wave therapy versus placebo [5,6,8,13,14,15,17,18,25,31], five only used shock wave therapy [7,12,16,23,28], others compared them with other treatments, such as: mirror therapy [1], ultrasound [29], and radial versus focused shock waves [19], in comparison with pharmacological treatment with BTA [27].

In terms of methodological quality, according to the Jadad scale score [37], nine of the RCT studies had a score of 3, which is considered an acceptable methodological quality, and the other five had a score of 5, representing rigorous quality. The average score was 3.8. Items with the worst rating on the quality scale were those related to blinding. The others were different types of study designs, presenting lower levels of evidence according to the pyramid of quality of scientific evidence [38]. These notably included a non-randomized controlled study, nine quasi-experimental pre–post studies, a case-control study, and a series of cases. Regarding the pretest–posttest quasi-experimental studies, the risk of bias was low (84%) according to the BAQA tool, with an average score of 9.2 points (out of 11) [39].

The criteria for inclusion of the different articles used for this work were mainly related to suspension of BTA prior to the study, motor impairment, and walking unaided (Table 1).

Regarding the protocol used, more than half of the studies did not specify if they used radial or focused shock waves in the intervention group. Of those that specified, only one study indicated that it used focused shock waves [19]. The protocols varied among the different authors: the total number of sessions ranged from 1 session to 20 sessions; the treatment times ranged from 1 week to 3 months; the shots administered ranged from 500 to 4000; the energy ranged from 0.03 mJ/mm^2^ −1.5 bar to 3.5 bar; the frequency ranged from 4 Hz to 10 Hz; the areas of study also varied depending on the objective of each author, as shown in Table 2.

### 3.4. Results of the Studies Included in the Review

**Figure 3:**
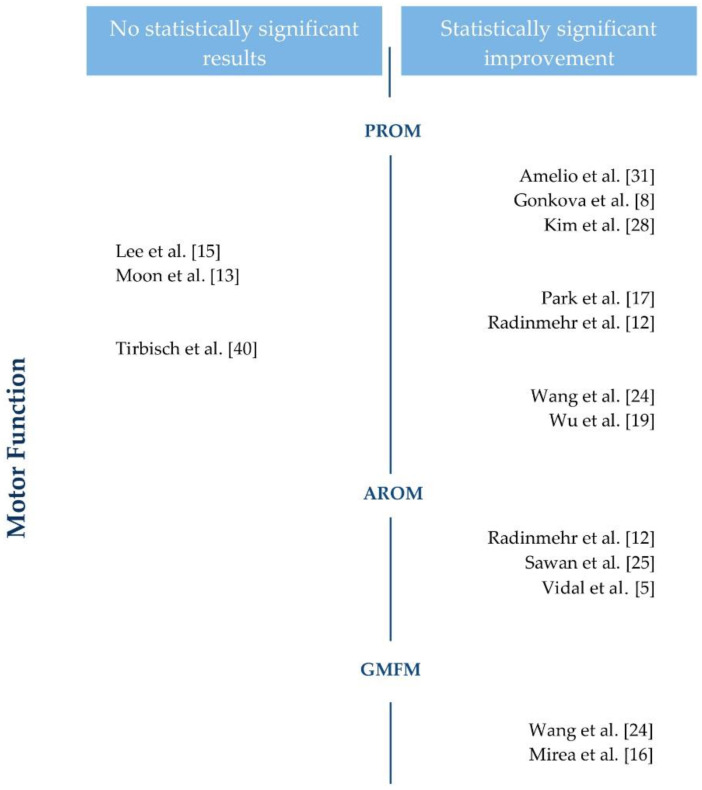
Motor function results of the studies included in the review. PROM: passive range of movement. AROM: active range of movement. GMFM: gross motor function classification system.

**Figure 4:**
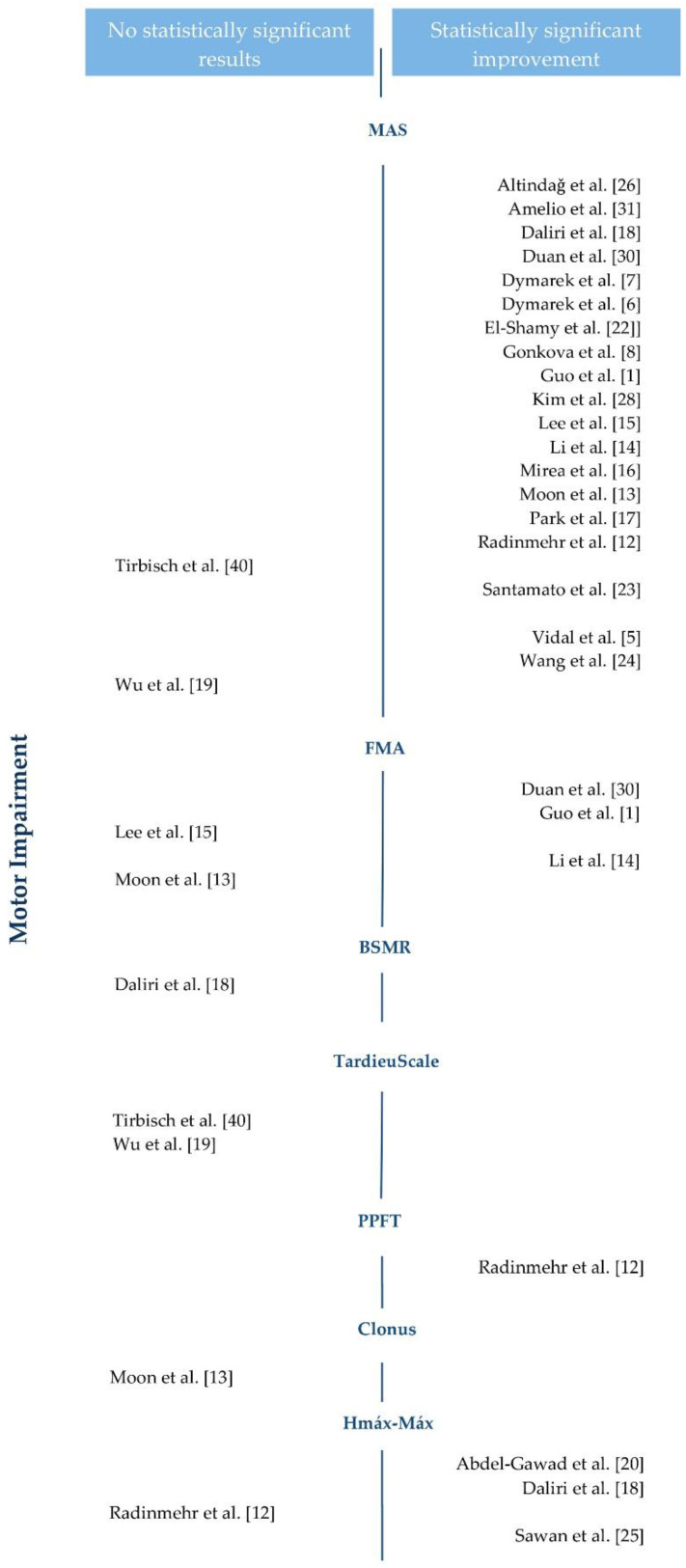
Motor impairment results of the studies included in the review. MAS: Modified Ashworth Scale. FMA: Fugl-Meyer. BMRS: Brunnstrom stages of motor recovery. PPFT: passive plantar flexor torque.

The 25 original studies included in this review use shock wave therapy on patients with spasticity to evaluate its effect on motor function, motor impairment, pain, and functional independence. Figure 3 and Figure 4 show the results regarding motor function and motor impairment.

Regarding motor function, 10 of the 15 studies found statistically significant improvements using different tools: passive range of movement (PROM) [8,12,13,15,17,19,24,27,28,29,31,40], active range of movement (AROM) [5,12,25,29], and the gross motor function classification system [16,24]. The other five studies observed positive changes without reaching differences [13,15,39], or non-inferiority versus the comparator [27,29].

Regarding motor impairment, 21 of the 25 studies that examined it reported statistically significant results using different tools: the modified Ashworth scale (MAS) [1,5,6,7,8,12,13,14,15,16,17,18,19,22,23,24,26,27,28,29,30,31,40], Fugl-Meyer (FMA) [1,13,14,15,27,30], the Hmax–Mmax ratio [12,18,20,25,29], and the passive plantar flexor torque (PPFT) [12,29], while the rest did not show superiority in relation to any of the therapies under comparison.

Pain was only evaluated by two studies [16,28], and both found significant differences, both using the questionnaire on pain caused by spasticity (QPS) and the visual analogue scale (VAS).

Finally, the level of functional independence of the patient with spasticity was evaluated in 10 of the 25 studies included, using different tools: gait analysis [20,22], dynamometric isocentric parameters [13], timed 10 m walk test [19,25], timed up and go test (TUG) [12,29], plantar contact area [19], and podobarometry [8,31]. The results were significant, and improvements in functional independence were identified in all but two studies [19,29].

At the same time, some authors used electrodiagnostic techniques to show the results obtained with shock waves; they observed statistically significant results through different techniques, such as electromyography (EMG) [6,7], and evaluated trophic conditions of the tissue with thermography (IRT) [6,7] or through echography [15].

## 4. Discussion

This systematic review includes 25 studies comparing shock waves with other therapies, such as conventional rehabilitation, mirror therapy, ultrasound, BTA, or a placebo. A total of 866 participants with spasticity, mainly related to stroke or CP, were included. The participants also presented prior suspension of BTA, motor impairment, and a need for a greater or lesser level of assistance with walking. The results obtained through the variables studied, such as motor function, motor impairment, pain, functional independence, and electrodiagnostic techniques, suggest that shock wave therapy could reduce levels of spasticity regardless of the age of the participants and the type of injury.

The mechanism of action of shock wave therapy could be related to a direct modulation of the rheological properties of the spastic muscle [16,17,19,20,31]. The mechanical shock (vibration) of the shock wave can break the functional link between the actin and the myosin, reducing the rigidity of the connective tissue of the spastic muscle [19,26,27]. Furthermore, it was hypothesized that the waves can dilate the blood vessels through enzymatic and non-enzymatic synthesis of nitric oxide (NO). NO is involved in neuromuscular junction formation in the peripheral nervous system and in physiological functions of the central nervous system, such as neurotransmission, memory, and synaptic plasticity. The synthesis of NO subsequently induces neovascularization, increasing the blood supply to the tissue and modulating the secretion of interleukins, thus regulating inflammation and activating the growth factor in the spastic muscle [17,20,22,27,31].

Different studies apply the shock waves with different protocols, although they all apply a minimum of 500 pulses per area of study to induce a cellular stimulation effect [23], and the same periods of intervention between groups. Eleven studies included in the review list the type of wave used; only one stated that it used focused waves [19], which is consistent with the evidence that radial waves cover a larger treatment area, and require a less precise focus, without the need for local anaesthesia, and at a lower cost [8]. Defining an optimal protocol for application of this therapy for spasticity is a clinical challenge that requires specific studies to be carried out to allow it to be standardized. The evidence shows that there is no relationship between the number of shots administered and the therapeutic effect for reduction of levels of spasticity [23]. Furthermore, the studies included applied therapy on different muscles: the soleus and gastrocnemius [8,12,13,15,16,17,19,20,22,23,24,25,26,29,31,40], flexor carpi radialis, and flexor carpi ulnaris [6,7,14,16,18,27], biceps brachii [16,27,30], intrinsics, and finger flexors [1,14], and the subscapularis [28], giving rise to positive results, regardless of the area treated.

The results of this review show a clear positive impact of show waves on motor function, motor impairment, functional independence, and the resulting improvement in activities in daily life, regardless of the form of measurement of those variables. The electrodiagnostic findings also suggest a reduction in bioelectric activity at rest and an improvement in trophic conditions thanks to the shock waves. Pain is one of the most common symptoms among people with spasticity, although it was only studied in two of the 25 studies included. The evidence indicates how the effects of the shock waves could reduce localized ischemia in areas of muscle shortening, reducing in turn the secretion of various substances that induce pain, and inhibiting inducing of pain due to stimulation of the nociceptors of the affected muscle; thus, increasing the range of joint motion and, as a result, quality of life [16,28]. Regarding the number of sessions, studies applying protocols with a large number of sessions observed better results on motor function. Specifically, a total of 1500 pulses were applied in the middle of the belly of the muscle, one session per week for three–six weeks [17,25].

This study has limitations, notably the heterogeneity of the shock wave protocols and the measuring tools, and the existence of original studies with non-randomised designs. The design of the study could have a very minor influence in the results because 22 of the 25 studies included in the review found statistically significant improvements for at least one outcome variable related to spasticity, regardless of the design of the study. Two RCTs included in the review [27,29] found that shock wave therapy showed non-inferiority compared to the alternative, and just one RCT (with a sample size of 8 participants) [40] did not show statistically significant improvements. However, the fact that significant improvements were found in the majority of the studies, despite said heterogeneity, supports the usefulness of shock waves in spasticity, regardless of the protocol and the form of evaluation. Furthermore, this study demonstrates the effectiveness of shock waves is a new possible kind of treatment along other treatments.

Currently, the treatment techniques chosen for handling spastic patients vary greatly. One of the techniques used is BTA, which has proven its efficacy in improving muscular spasticity [41], as it reduces hyperactivity by acting on the cytosol of the nerve endings, and it inhibits release of acetylcholine in the neuromuscular junctions [21]. Evidence [1,2,3,9,10,21,42] suggests that complementary therapies can improve results after injection of BTA. However, some of these therapies imply a considerable risk of unwanted effects in the long-term: antispasmodic medication administered orally can induce weakness in healthy muscles, chemical neurolysis can cause dysaesthesia, repeated injections of BTA can stimulate formation of antibodies [14,27], etc. Therefore, one of the advantages of shock wave therapy is its effectiveness, with a low-risk of side effects, as it is a non-pharmacological and non-invasive technique [1,6,14,24] for reducing spasticity, either as a monotherapy or together with medication and/or other physiotherapy techniques.

## 5. Conclusions

Shock wave therapy shows positive results as an alternative for treatment of spasticity, to improve motor function and motor impairment, to reduce pain, and to improve functional independence, even from a single session, and applied independently of BTA. However, given the heterogeneity of the shock wave protocols employed (in terms of the number of sessions, duration, shots, energy, and frequency), further studies are required to determine the conditions under which the best results can be obtained.

## Figures and Tables

**Table 1:** Descriptive characteristics and results of the included studies.

Authors/Year	Study Design	Participants	Intervention	Comparative	Outcomes Variable (Tool)
AbdelGawad et al. (2015) [20]	Randomized Controlled Trial	Dx: CP Age (mean ± standard deviation years): (I) 5.75 ± 0.51 (C) 5.83 ± 0.34 Sex: 60% F, 40% M Inclusion criteria: No BTA, MAS 1-2, standing	*n* = 15 Shock wave therapy and conventional rehabilitation programme	*n* = 15 conventional rehabilitation programme	Motor impairment (Hmax–Mmax) Functional independence (gait analysis)
Altindaǧ et al. (2014) [26]	Case series	Dx: Stroke, CP, SPI Age (years): 36.6 ± 23.37 Sex: 44% F, 56% M Inclusion criteria: no BTA, MAS > 2	*n* = 9 Shock wave therapy and conventional rehabilitation programme		Motor impairment (MAS)
Amelio et al. (2010) [31]	Quasi-experimental pre-post	Dx: CP Age (years): 5.83 ± 2.31 Sex: 50% F, 50% M Inclusion criteria: no BTA, gait	*n* = 12 1st session placebo and 2nd session shock wave therapy		Motor Function (PROM) Motor impairment (MAS) Functional independence (podobarometry)
Daliri et al. (2015) [18]	Quasi-experimental pre-post	Dx: Stroke Age (years): 54.4 ± 9.4 Sex: 20% F, 80% M Inclusion criteria: no BTA, Stroke > 6 months	*n* = 15 1st session placebo and 2nd session shock wave therapy		Motor impairment (MAS, BMRS, Hmax-Mmax)
Duan et al. (2016) [30]	Randomized Controlled Trial	Dx: Stroke Age (years) (I) 48.29 ± 12.30 (C) 50.67 ± 14.27 Sex: 48% F, 52% M Inclusion criteria: no BTA	*n* = 24 Shock wave therapy and conventional rehabilitation programme	*n* = 24 conventional rehabilitation programme	Motor impairment (MAS, FMA)
Dymarek et al. (b/2016) [7]	Quasi-experimental pre-post	Dx: Stroke Age (years): 63.15 ± 12.60 Sex: 65% F, 35% M Inclusion criteria: no BTA, MAS > 1+, Stroke > 9 months	*n* = 20 Shock wave therapy		Motor impairment (MAS) Electrodiagnostic (EMG, IRT)
Dymarek et al. (a/2016) [6]	Randomized Controlled Trial	Dx: Stroke Age (years) (I) 61.43 ± 12.74 (C) 60.87 ± 9.51 Sex: 43% F, 57% M Inclusion criteria: no BTA, MAS > 1+, Stroke > 9 months	*n* = 30 Shock wave therapy	*n* = 30 Placebo	Motor impairment (MAS) Electrodiagnostic (EMG, IRT)
El-Shamy et al. (2014) [22]	Randomized Controlled Trial	Dx: CP Age (years) (I) 6.93 ± 0.8 (C) 6.8 ± 0.77 Sex: 40% F, 60% M Inclusion criteria: No BTA, independent gait	*n* = 15 Shock wave therapy and conventional rehabilitation programme	*n* = 15 conventional rehabilitation programme	Motor impairment (MAS) Functional independence (gait analysis)
Gonkova et al. (2013) [8]	Quasi-experimental pre-post	Dx: CP Age (years): 4.84 ± 3.11 Sex: 36% F, 64% M Inclusion criteria: no BTA	*n* = 25 1st session placebo and 2nd session radial shock wave therapy		Motor Function (PROM) Motor impairment (MAS) Functional independence (podobarometry)
Guo et al. (2019) [1]	Randomized Controlled Trial	Dx: Stroke Age (years) (I–A) 66.79 ± 11.02 (I–B) 67.15 ± 11.23 (I–C) 68.72.0 ± 10.56 (C) 69.72 ± 11.13 Sex: 44% F, 56% M Inclusion criteria: no BTA, MAS > 1, <4, Stroke > 6 months	Group A: *n* = 30 Shock wave therapy and conventional rehabilitation programme Group B: *n* = 30 Mirror therapy and conventional rehabilitation programme Group C: *n* = 30 Mirror therapy, shock wave and conventional rehabilitation programme	*n* = 30 conventional rehabilitation programme	Motor impairment (MAS, FMA)
Kim et al. (2013) [28]	Quasi-experimental pre-post	Dx: stroke Age (years): 55.4 ± 13.2 Sex: 42% F, 58% M Inclusion criteria: no BTA, MAS > 1, Stroke > 9 months	*n* = 57 Radial shock wave therapy and conventional rehabilitation programme		Motor function (PROM) Motor impairment (MAS) Pain (VAS)
Lee et al. (2019) [15]	Randomized Controlled Trial	Dx: stroke Age (years): (I) 50.89 ± 8.81 (C) 44.11 ± 4.07 Sex: 11% F, 89% M Inclusion criteria: No BTA	*n* = 9 Radial shock wave therapy	*n* = 9 Placebo	Motor function (PROM) Motor impairment (MAS, FMA) Electrodiagnostic (echography)
Li et al. (2016) [14]	Randomized Controlled Trial	Dx: stroke Age (years): (I–A) 55.35 ± 3.05 (I–B) 56.80 ± 3.00 (C) 55.95 ± 2.64 Sex: 31% F, 69% M Inclusion criteria: no BTA, MAS > 1, Stroke > 9 months	Group A: *n* = 20 3 sessions of radial shock wave therapy Group B: *n* = 20 1 session of shock wave therapy	*n* = 20 Placebo	Motor impairment (MAS, FMA)
Mirea et al. (2014) [16]	Quasi-experimental pre-post	Dx: CP Age (months): 99.57 ± 53.74 Sex: 41% F, 59% M Inclusion criteria: no BTA, MAS 1−3	*n* = 63 Shock wave therapy		Motor function (GMFM) Motor impairment (MAS) Pain (QPS)
Moon et al. (2013) [13]	Quasi-experimental pre-post	Dx: stroke Age (years): 52.6 ± 14.9 Sex: 43% F, 57% M Inclusion criteria: no BTA, MAS > 1+, Stroke > 1 month.	*n* = 30 1 placebo session and 3 sessions of shock wave therapy		Motor function (PROM) Motor impairment (MAS, FMA, clonus) Functional independence (dynamometric isocentric parameters)
Park et al. (2015) [17]	Randomized Controlled Trial	Dx: CP Age (years): (I) 7.0 ± 3.1 (C) 6.8 ± 2.3 Sex: 41% F, 59% M Inclusion criteria: no BTA, gait	*n* = 6 3 sessions of shock wave therapy and conventional rehabilitation programme	*n* = 6 1 shock wave session and 2 placebo sessions and conventional rehabilitation programme	Motor function (PROM) Motor impairment (MAS)
Radinmehr et al. (2016) [12]	Randomized Controlled Trial	Dx: stroke Age (years): 59 ± 13 Sex: 41% F, 59% M Inclusion criteria: no BTA, independent gait, MAS > 1, Stroke > 1 month	*n* = 12 Radial shock wave therapy		Motor function (PROM, AROM) Motor impairment (MAS, PPFT, Hmax–Mmax) Functional independence (TUG)
Radinmehr et al. (2019) [29]	Randomized Controlled Trial	Dx: stroke Age (years): (I) 56.0 ± 12.3 (C) 56.2 ± 8.4 Sex: 40% F, 60% M Inclusion criteria: no BTA, gait, MAS > 1, Stroke > 1 month	*n* = 16 Radial shock wave therapy	*n* = 16 Ultrasounds	Motor function (PROM, AROM) Motor impairment (MAS, PPFT, Hmax–Mmax) Functional independence (TUG)
Santamato et al. (2014) [23]	Quasi-experimental pre-post	Dx: stroke Age (years): 57.6 ± 10.8 Sex: 34% F, 66% M Inclusion criteria: no BTA, MAS > 1 <4	*n* = 23 Shock wave therapy		Motor impairment (MAS)
Sawan et al. (2017) [25]	Quasi experimental	Dx: stroke Age (years): (I) 50.6 ± 6.7 (C) 84.8 ± 5.9 Inclusion criteria: no BTA, MAS 1−2	*n* = 20 Shock wave therapy and conventional rehabilitation programme	*n* = 20 Placebo and conventional rehabilitation programme	Motor function (AROM) Motor impairment (Hmax–Mmax) Functional independence (Timed 10 m walk test)
Tirbisch et al. (2015) [40]	Randomized Controlled Trial	Dx: stroke Inclusion criteria: no BTA, MAS > 1+	*n* = 4 Radial shock wave therapy and conventional rehabilitation programme	*n* = 4 conventional rehabilitation programme	Motor function (PROM) Motor impairment (MAS, Tardieu Scale)
Vidal et al. (2011) [5]	Randomized Controlled Trial	Dx: CP Age (years): 31 Sex: 20% F, 80% M Inclusion criteria: no BTA	*n* = 5 Radial shock wave therapy on spastic agonists. *n* = 5 Radial shock wave therapy on spastic agonists and antagonists	*n* = 5 Placebo	Motor function (AROM) Motor impairment (MAS)
Wang et al. (2016) [24]	Case-control	Dx: CP Age (months): (I) 26.9 ± 13.1 (C) 27.0 ± 14.2 Sex: 33% F, 67% M Inclusion criteria: no BTA, MAS 1−4	*n* = 34 Shock wave therapy and conventional rehabilitation programme	*n* = 32 Conventional rehabilitation programme	Motor function (PROM, GMFM) Motor impairment (MAS)
Wu et al. (a/2018) [19]	Randomized Controlled Trial	Dx: stroke Age (years): (I) 59.6 ± 11.3 (C) 60.3 ± 9.9 Sex: 41% F, 59% M Inclusion criteria: no BTA, gait, MAS 1-4, Stroke > 6 months	*n* = 16 Radial shock wave therapy	*n* = 15 Focused shock wave therapy	Motor function (PROM) Motor impairment (MAS, Tardieu Scale) Functional independence (Timed 10 m walk test, plantar contact area)
Wu et al. (b/2018) [27]	Randomized Controlled Trial	Dx: stroke Age (years): (I) 60.0 ± 11.1 (C) 62.0 ± 10.2 Sex: 33% F, 67% M Inclusion criteria: no BTA, Stroke > 6 months	*n* = 21 Shock wave therapy	*n* = 21 BTA	Motor function (PROM) Motor impairment (MAS, FMA)

Dx: diagnosis; CP: Cerebral Palsy; F: Female; M: Male; BTA: Botulinum toxin; MAS: Modified Ashworth Scale, SCI: Spinal Cord Injury; PROM: passive range of movement; BMRS: Brunnstrom stages of motor recovery; FMA: Fugl-Meyer assessment; EMG: electromyography; IRT: thermography; VAS: Visual analogic Scale; GMFM: gross motor function classification system; QPS: questionnaire of pain caused by spasticity; AROM: active range of movement; TUG: Timed up and go test; PPFT: passive plantar flexor torque.

**Table 2:** Shock wave protocols used for studies included.

Authors	Type of Shock Waves	Number of Sessions	Treatment Time	Number of Shots	Energy	Frequency	Anatomic Area
AbdelGawad et al. [20]	Not specified	3	1 week	2100	0.32 mJ/mm^2^		Soleus and gastrocnemius
Altindaǧ et al. [26]	Not specified	3	2 weeks	2000	0.1 mJ/mm^2^ 2 bar		Soleus and gastrocnemius
Amelio et al. [31]	Not specified	1	8 weeks	1500	0.03 mJ/mm^2^ 1.5 bar		Soleus and gastrocnemius
Daliri et al. [18]	Not specified	1	2 weeks	1500	0.03 mJ/mm^2^ 1.5 bar		Flexor carpi radialis and flexor carpi ulnaris
Duan et al. [30]	Not specified		1 week				Biceps brachii
Dymarek et al. (b/2016) [7]	Not specified	1	1 week	1500	0.03 mJ/mm^2^ 1.5 bar	4 Hz	Flexor carpi radialis and flexor carpi ulnaris
Dymarek R et al. (a/2016) [6]	Not specified	1	1 week	1500	0.03 mJ/mm^2^ 1.5 bar	5 Hz	Flexor carpi radialis and flexor carpi ulnaris
El-Shamy et al. [22]	Not specified	12	3 months	1500	0.03 mJ/mm^2^ 1.5 bar	5 Hz	Soleus and gastrocnemius
Gonkova et al. [8]	Radials	1	5 weeks	1500	0.03 mJ/mm^2^ 1.5 bar	5 Hz	Soleus and gastrocnemius
Guo et al. [1]	Not specified	20	4 weeks	2000	2–3 bar	8 Hz	Intrinsic and flexor digitorum
Kim et al. [28]	Radials	5	2 weeks	3000	0.63 mJ/mm^2^ 1.6 bar	8 Hz	Subscapularis
Lee et al. [15]	Radials	1	1 week	2000	0.1 mJ/mm^2^ 2 bar	4 Hz	Soleus and gastrocnemius
Li et al. [14]	Radials	3	3 weeks	1500 4000	3.5 bar 3 bar	5 Hz 5 Hz	Flexor carpi radialis and flexor carpi ulnaris, intrinsic and flexor digitorum
Mirea et al. [16]	Not specified	3	3 weeks	500	0.15 mJ/mm^2^ 1.5 bar	10 Hz	Soleus and gastrocnemius, biceps brachii, Flexor carpi radialis and flexor carpi ulnaris
Moon et al. [13]	Not specified	3	4 weeks	1500	0.089 mJ/mm^2^	4 Hz	Soleus and gastrocnemius
Park et al. [17]	Not specified	1/3	1 week	1500	0.03 mJ/mm^2^ 2.5 bar	4 Hz	Soleus and gastrocnemius
Radinmehr et al. (2016) [12]	Radials	1	1 week	2000	0.34 mJ/mm^2^ 1 bar	5 Hz	Soleus and gastrocnemius
Radinmehr et al. (2019) [29]	Radials	1	1 week	2000	0.34 mJ/mm^2^ 1 bar	5 Hz	Soleus and gastrocnemius
Santamato et al. [23]	Not specified	1	1 week	1500	0.1 mJ/mm^2^ 2 bar		Soleus and gastrocnemius
Sawan et al. [25]	Not specified	6	6 weeks	1500	0.34 mJ/mm^2^ 1 bar	5 Hz	Soleus and gastrocnemius
Tirbisch et al. [40]	Radials	9	3 weeks		0.03 mJ/mm^2^	10 Hz	Soleus and gastrocnemius
Vidal et al. [5]	Radials	3	1 week	4000	0.10 mJ/mm^2^ 2 bar	8 Hz	Agonists and antagonists
Wang et al. [24]	Radials	12	3 months	3000	0.03 mJ/mm^2^ 0.6 bar	8 Hz	Soleus and gastrocnemius
Wu et al. (a/2018) [19]	Radials Focused	3 3	1 week 1 week	3000 3000	2 bar 0.10 mJ/mm^2^	5 Hz 5 Hz	Soleus and gastrocnemius Soleus and gastrocnemius
Wu YT et al. (b/2018) [27]	Not specified	3	1 week	3000	3.5 bar	5Hz	Flexor carpi radialis and flexor carpi ulnaris and biceps brachii.

## Data Availability

The data presented in this study are available on request from the corresponding author.
